# Usefulness of magnetic resonance enterography in detecting signs of sacroiliitis in young patients with inflammatory bowel disease

**DOI:** 10.1186/s12969-020-00433-w

**Published:** 2020-06-03

**Authors:** Teresa Giani, Azzurra Bernardini, Massimo Basile, Marco Di Maurizo, Anna Perrone, Sara Renzo, Viola Filistrucchi, Rolando Cimaz, Paolo Lionetti

**Affiliations:** 1 Anna Meyer Children’s Hospital, Florence, Italy; 2 Department of Medical Biotechnology, University of Siena, Siena, Italy; 3 Department of Clinical Sciences and Community Health and Research Center for Adult and Rheumatic Diseases, University of Milan, Milan, Italy; 4 Neurofarba Department, University of Florence, Florence, Italy

**Keywords:** Sacroiliitis, Pediatric, Magnetic resonance imaging (MRI), Magnetic resonance enterography (MRE), Inflammatory bowel disease (IBD)

## Abstract

**Background:**

Arthritis is often an underestimated extraintestinal manifestation in pediatric inflammatory bowel disease (IBD), including sacroiliitis, whose early signs are well detectable at magnetic resonance imaging (MRI). Magnetic resonance enterography (MRE) is an accurate imaging modality for pediatric IBD assessment.

We studied the possibility to detect signs of sacroiliac inflammation in a group of children with IBD who underwent MRE for gastrointestinal disease evaluation.

**Methods:**

We retrospectively reviewed MRE scans performed in pediatric patients with IBD. We looked for signs of sacroiliitis taking the ASAS (Assessment of SpondyloArthritis international Society) criteria as a model. Presence of bone marrow edema (using T2W sequences with fat suppression), diffusion restriction in Diffusion Weighted Imaging (DWI) or Diffusion Weighted Imaging with Background Suppression (DWIBS), and dynamic contrast enhancement were evaluated. Each SI joint was divided into 4 quadrants: upper iliac, lower iliac, upper sacral, and lower sacral. Two blinded observers with experience in pediatric and skeletal imaging independently evaluated the images. Cases upon which there was a disagreement were evaluated by the two reviewing radiologists and a third radiologist with similar experience together.

**Results:**

We enrolled 34 patients (24 males and 10 females, with mean age at scanning 14.3 years, median 15.3 years; 2 affected by ulcerative colitis, 32 by Crohn’s disease) for a total of 59 examinations performed at the time of their first diagnosis or at symptom exacerbations. No patient complained of musculoskeletal symptoms, neither had pathological findings at articular examination. At the time of MRE 25 patients were under treatment for their IBD. Five patients had radiological signs of SI inflammation at MRE, albeit of mild degree. All patients with SI joint edema also had a restricted diffusion in DWIBS or DWI and almost everyone had contrast media uptake.

**Conclusions:**

Sacroiliitis is one of the extraintestinal manifestation associated with IBD; it is often asymptomatic and clinically underdetected, with an unrelated progression with respect to the underlying IBD. MRE offers the possibility to study SI joints in young patients with IBD who undergo MRE for the investigation of their intestinal condition. Furthermore, we observed that gadolinium enhancement does not improve diagnostic specificity in sacroiliiitis detection.

## Background

Inflammatory bowel diseases (IBD), as Crohn’s disease (CD), and Ulcerative Colitis (UC), are a group of chronic and relapsing inflammatory conditions often diagnosed in patients younger than 20 years of age [1]. In addition to bowel symptoms, patients with IBD often present extraintestinal complications, such as arthritis, eye disorders, skin problems, kidney and liver disease [2–4].

The most common extraintestinal complication of these disorders is arthritis, which has been reported in 7 to 21% of children with IBD [5]. The causes of IBD and the concomitant arthritis remain unclear, although immunopathological overlap between gut inflammation and spondyloarthropathies has been demonstrated. Intestinal inflammation is believed to be heavily involved in the pathogenesis of spondyloarthropathy [6]. Two patterns of joint inflammation are described: peripheral polyarthritis and, less commonly, involvement of the sacroiliac (SI) joints and axial skeleton. Whereas the peripheral arthritis reflects the activity and course of the gastrointestinal (GI) inflammation, sacroiliitis may show poor correlation to the activity of gut disease [7], and may also be asymptomatic [5, 8–10]. In addition, no laboratory test is considered reliable for diagnosis and management of these conditions [11].

Althought infrequent, the SI involvement in the course of pediatric IBD is often asymptomatic and clinically underdetected; in addition, the inflammatory damage at SI joint may progress regardless of the control of the underlying IBD. Magnetic resonance imaging (MRI) is very sensitive in assessing subclinical sacroiliitis by identifying bone marrow edema as the primary sign of SI inflammation [12, 13], before any X-Ray sign is identifiable [14–16].

On the other hand, MR enterography (MRE) is the current gold standard for imaging to assess IBD intestinal disease activity [17].

We studied the ability of MRE performed in a group of children affected by IBD for bowel evaluation in order to identify signs of SI inflammation.

In recent years only few studies have been conducted on adult patients with IBD in order to define the role of MRE in assessing sacroiliitis [18, 19], and the data available on pediatric patients have been obtained by MRI [20, 21].

## Materials and methods

### Patients

This is a retrospective study based on the review of clinical and imaging data of pediatric patients who underwent MRE between March 2010 and December 2018 for a suspicion of IBD or for disease follow-up at Meyer Children’s University Hospital of Florence, Italy.

Some patients underwent multiple examinations.

First, the feasibility of sacroiliac joints study on MRE examinations was evaluated, since a tailored sequence for this analysis was not normally included in standard MRE protocol*.*

The inclusion criteria were: (i) presence of T2 SPAIR or STIR sequences on coronal or axial plane; (ii) presence of DWI or DWIBS on coronal or axial plane; (iii) presence of T1W post gadolinium or dynamic contrast enhancement sequences; (iv) good diagnostic quality of these sequences.

### Magnetic resonance Enterography protocol

MRE examinations were performed on 1.5 T (Achieva; Philips Medical System, Best, The Netherlands) or 3 T (Achieva, Philips Medical System) MRI scanners with a phased-array body coil, as previously described [17]. Patients were asked to follow a 4 days’ residue- free diet and a 6-h fast. Before the examination they were given a hyperosmotic oral aqueous solution mixed with dilute sorbitol at 70% (ACEF Spa, Piacenza, Italy) to be taken over a period of 40 to 45 min: the first 50 mL of sorbitol in 200 mL of water in 15 to 20 min and other 50 mL of sorbitol in 300 mL of water in 20 to 25 min. During the MRE patients were in the prone position and if not contraindicated a body weight-based dose of scopolamine (Buscopan; Boehringer Ingelheim, Ingelheim, Germany) was administered intravenously before the contrast agent to obtain bowel relaxation and peristalsis reduction. Gadoteratemeglumine (Dotarem, Guerbet, Villepinte, France, 0.5 mmol/mL) was used for all patients at the recommended dose of 0.2 mL/kg, followed by a saline flush. Standard MRE protocol is shown in Table 1.

**Table 1:** MRI protocol used at our institution for patients with Inflammatory Bowel Disease

Parameters	TR, ms	TE, ms	TI, ms	Matrix	B values	Slice Thickness,mm
cor dyn BTFE	4.7	2.4	–	228 × 224	–	10
ax BTFE F-B	3.5	1.7	–	192 × 159	–	3
cor BTFE F-B	3.8	1.9	–	288 × 188	–	3
cor T2 SPAIR	1060.5	70	–	244 × 188	–	3
ax DWI	2270.7	68.4	–	96 × 96	0–500-1000	4
ax DWIBS	9450.8	54.3	220	104 × 98	–	6
ax T2	856.9	70	–	208 × 158	–	3
cor T1 TFE SPIR	10	2.3	–	200 × 228	–	10
ax dyn THRIVE	3.1	1.5	–	172 × 172	–	3.6
cor T1 TFE SPIR mdc	10	2.3	–	228X168	–	5

### Image analysis

**Fig. 1:**
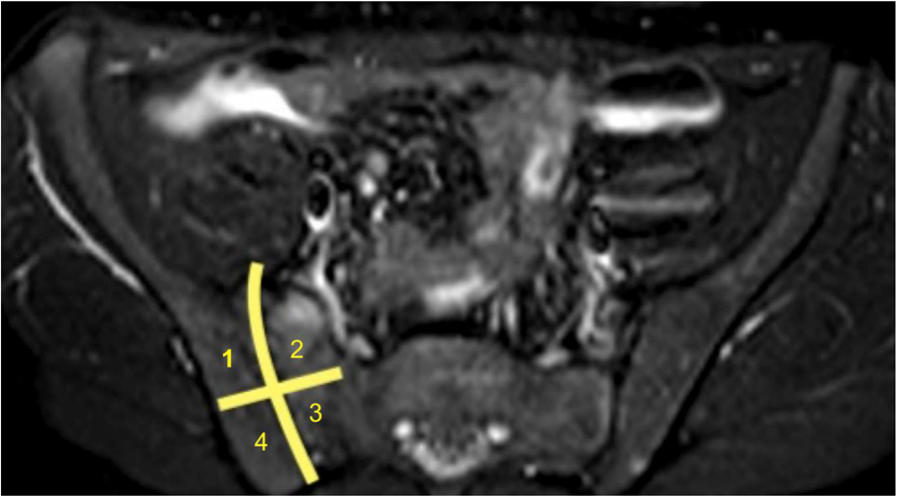
Subdivision of SI joint into 4 quadrants: 1 (upper iliac), 2 (upper sacral), 3 (lower sacral) and 4 (lower iliac)

Two blinded observers with experience in pediatric and skeletal imaging (AP, MDM) independently evaluated the images. Before reading the MRE, a consensus about the definition of inflammatory lesions in SI joints was reached. MRI signs of sacroiliitis in adults are described by the ASAS criteria [22], but at the moment, no such definition of a positive MRI for sacroiliitis exists in children with juvenile spondyloarthritis [23]. For this reason, and for the retrospective nature of this study, where targeted sequences for SI joints analysis on MRE lacked, we decided to evaluate the presence of bone marrow edema (using T2W sequences with fat suppression, SPAIR), as a defining sign of sacroiliitis. In addition, the presence of diffusion restriction in Diffusion Weighted Imaging (DWI) or Diffusion Weighted Imaging with Background Suppression (DWIBS), and dynamic contrast enhancement were evaluated. Each SI joint was divided into 4 quadrants: upper iliac, lower iliac, upper sacral, and lower sacral (Fig. 1). Cases upon which there was a disagreement were reevaluated together by the two reviewing radiologists and a third radiologist (MB) with similar experience.

Demographics, IBD features, clinical, radiological and laboratory data were recorded in a dedicated Excel database. No ethics committee approval was deemed necessary, since by local regulations anonimyzed data were used.

## Results

We reviewed 128 MRE performed during the study period at our Radiological Unit.

Forty-six examinations did not meet inclusion criteria and were excluded since they did not have DWI sequences, which was initially optional in the MRE protocol, or sacroiliac joints were not included or only partially included in the examinations. Additionally, 23 examinations were excluded due to the poor diagnostic quality of the images needed for the sacroiliac joints analysis.

Thirty-four patients were therefore enrolled (24 males and 10 females, mean age at scanning 14.3 years, median 15.3 years) for a total of 59 examinations performed at the time of their first diagnosis or at symptoms exacerbations.

Two out of 34 patients were affected by UC, 32 by CD. Mean disease duration was 2.9 years, median 2.1 years. Clinical evaluation of the joints resulted negative in all patients and none complained of articular symptoms including back pain. At the time of MRE, 25 patients were under treatment: 14 were receiving immunosuppressants (methotrexate, azathioprine, 6-MP, thalidomide) or amynosalicilate (mesalazine), 6 were receiving biologic (anti-TNF) therapy, 3 were taking a combination of immunosuppressants and biologics, and 2 immunosuppressants associated with corticosteroids.

For all 59 MRE inter-reader agreement was good (Cohen’s kappa > 0.815). All cases of doubtful inflammatory sacroiliitis and discrepancy (*n* = 8) were resolved after discussion between the two reviewing radiologists and a third radiologist.

**Fig. 2:**
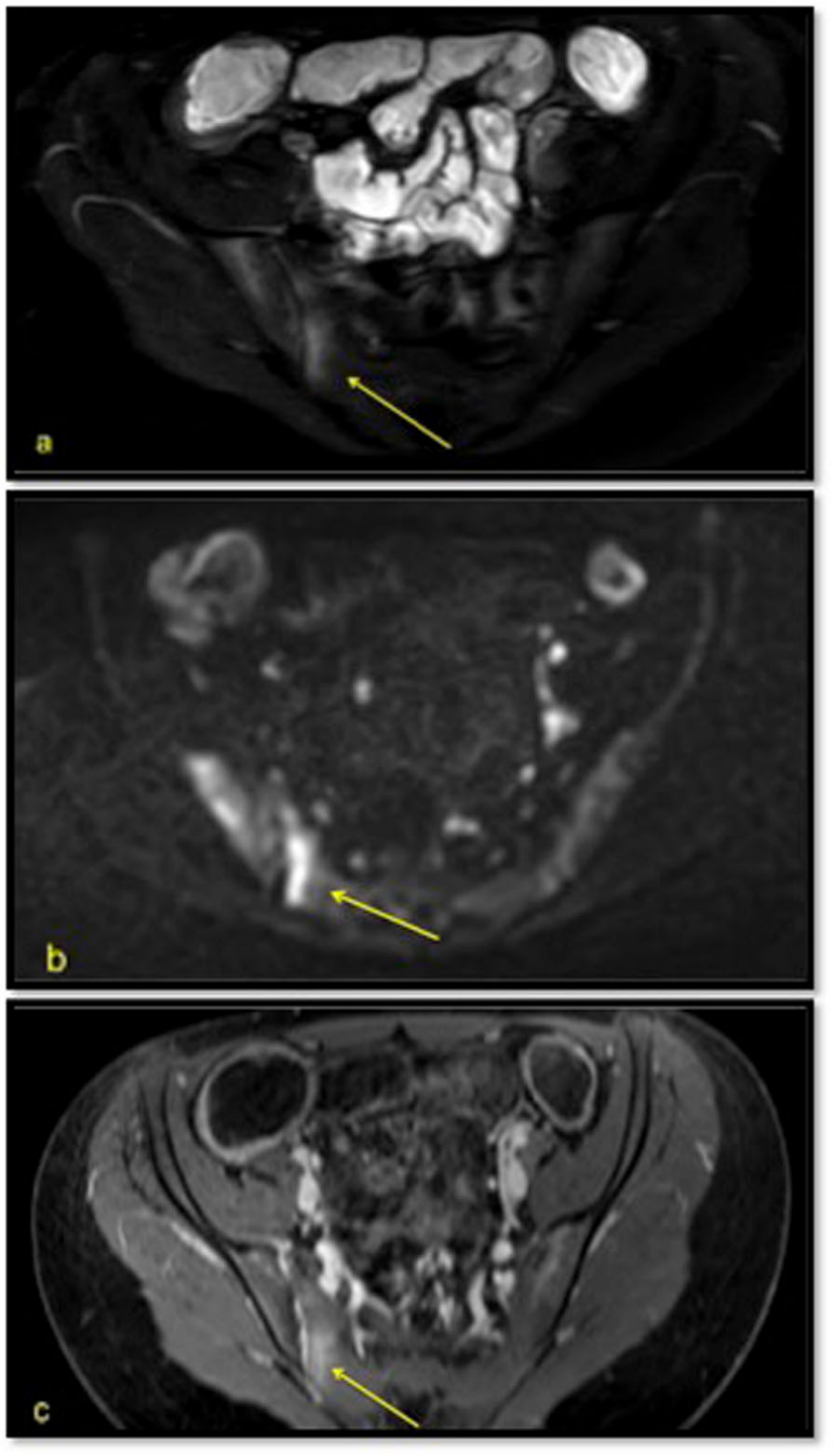
Axial T2 SPAIR sequence (**a**) and axial DWIBS sequence (**b**) show hyperintensity in the lower right sacral quadrants due to edema. In the same quadrant there is hyperintensity after contrast administration (**c**)

In 6 MRE scans (of 5 IBD patients), a monolateral slight degree of sacroiliitis was radiologically identified (Fig. 2). Five out of 6 MRE examination had positive findings in all sequences evaluated (T2 W, DWI/DWIBS and dynamic contrast enhancement), while 1 out of 6 had positive findings on T2 W, DWI/DWIBS without contrast enhancement. The characteristic of patients with sacroiliitis compared with patients without sacroiliitis on MRE are reported in Table 2. No significant differences between the two groups were seen.

**Table 2:** Characteristics of patients with sacroiliitis (YES) vs. without sacroiliitis (NO) at MRE

	NO		YES	
	*N* = 29		*N* = 5	
Characteristics	N	%	N	%
**IBD**				
UC	1	3.5	1	20
CD	28	96.5	4	80
**Gender**				
Male	19	65.5	5	100
Female	10	34.5	0	0
**Mean age at scanning** (years)	14.4	N/A	13.7	N/A
**Mean disease duration** (years)	3.6	N/A	2.9	N/A
**ESR** > 15 mm/h	16	55.2	4	80
**CRP** > 0.5 mg/dl	14	48.2	1	20
**Therapy at the time of MRE**				
IS	11	38	3	60
IS and biologicals	3	10.3	0	0
IS and CS	2	6.9	0	0
Biologicals	4	13.8	2	40
No therapy	9	31	0	0

*CD* Crohn’s disease, *UC* ulcerative colitis, *CRP* C-reactive protein, *CS* corticosteroids, *IBD* inflammatory bowel disease, *IS* immunosuppressants (methotrexate, azathioprine, *6-MP* thalidomide) or amynosalicylate (mesalazine), *MRE* magnetic resonance enterography, *N/A* not applicable

**Fig. 3:**
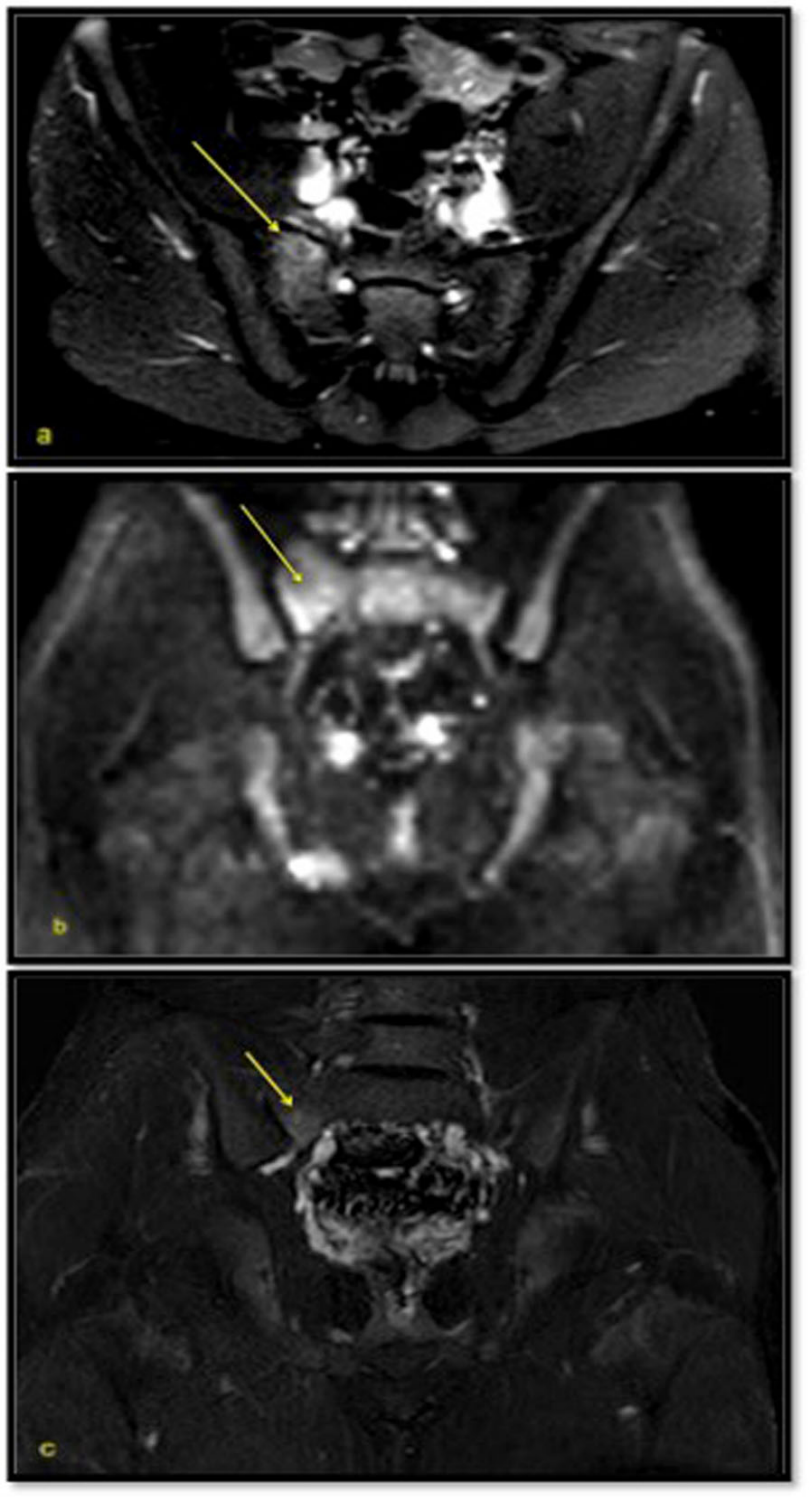
In the only patient discordant for gut and SI joint inflammation, axial T2 SPAIR sequence (**a**) and coronal DWIBS sequence (**b**) show hyperintensity in the upper right sacral quadrant due to edema. In the same quadrant there is hyperintensity after contrast administration (**c**). The MRE control was performed after 18 months of pharmacological treatment and showed the disappearance of intestinal signs of inflammation, while MR signs of sacroiliitis were still present

Four out of the five patients had no clinical, laboratory or radiological signs of intestinal inflammation at the time of MRE. One patient presented with signs of intestinal and sacroiliac inflammation at his first MRE. The MRE control performed after 18 months of pharmacological treatment (Infliximab) showed the disappearance of intestinal signs of inflammation, while MR signs of sacroiliitis were still present (Fig. 3).

## Discussion

To the best of our knowledge there are no data regarding the prevalence of sacroiliitis detected on MRE in pediatric IBD patients. Most studies report the prevalence of articular involvement (both peripheral and axial) in children with IBD being between 7 and 25% [2, 3, 12], more frequently in CD than UC patients [12, 24]. However, data have been obtained with standard MRI and not with MRE. Moreover, this wide range is probably due to the absence of clinical symptoms of many sacroiliitis diagnosed with MRI [12, 20]. If not diagnosed, sacroiliac inflammation is likely to progress with poor therapeutic prognosis [25–28] .

In our series, SI inflammation was present in about 15% of cases; of note, none of them had SI inflammatory symptoms, partially in agreement with the literature data [12, 20] which reported that up to 50% of IBD patients may be asymptomatic and only 24% of children with enthesitis-related arthritis complain of pain, stiffness or limitation of motion of the lumbosacral spine at presentation [5].

We think that it may be useful to evaluate SI inflammation in patients undergoing MRE for IBD, in order to reduce underdiagnoses in asymptomatic subjects. We also noted that all patients with SI joint edema had also a restricted diffusion in DWIBS or DWI, and almost everyone had contrast uptake, so the use of gadolinium could be avoided in pediatric patients as it does not contribute to the diagnosis, in agreement with previous studies on adult patients [22, 29]. This is a great benefit considering the necessity for patients to undergo several diagnostic procedure during the course of the disease.

We also suggest that in case of SI radiological abnormalities, even if with only mild edema, rheumatology team should be involved. This would implicate thorough physical examination, and a clinical follow-up. If needed, repeated scans might be needed and in case of clinical symptoms patient management might also be changed. This is an exploratory study and the number of patients included is small.

Limitations of our study are its retrospective nature and the fact that the orientation of the acquisition planes is not completely suitable for the SI joints analysis. In agreement with the recent literature [21], we suggest that it might be useful to add a sequence targeted to SI evaluation during a MRE. However, our study is the first of its kind and we think that our results could help to improve the management of extraintestinal manifestations of IBD and the therapeutic approach when both spondyloarthropathy and IBD are associated.

In conclusion, this pilot study demonstrates that MRE may be a good tool to detect early signs of SI inflammation, even in asymptomatic patients, but for a better evaluation of SI joints dedicated sequences may be necessary. Moreover, with the addition of such sequences not only the diagnostic accuracy could be improved but IBD patients could be spared the necessity of standard MRI even when symptomatic, since MRE are routinely used during follow-up.

## Data Availability

The datasets used and/or analysed during the current study are available from the corresponding author on reasonable request.

## Abbreviations

SI: Sacroiliac
IBD: Inflammatory bowel disease
MRE: Magnetic resonance enterography
ASAS: Assessment of SpondyloArthritis international Society
DWI: Diffusion Weighted Imaging
DWIBS: Diffusion Weighted Imaging with Background Suppression
GI: Gastrointestinal
MRI: Magnetic Resonance Imaging
CD: Crohn’s disease
UC: Ulcerative Colitis
